# Part-time hospitalisation and stigma experiences: a study in contemporary psychiatric hospitals

**DOI:** 10.1186/1472-6963-8-125

**Published:** 2008-06-10

**Authors:** Mieke Verhaeghe, Piet Bracke, Wendy Christiaens

**Affiliations:** 1 Department of Sociology, Ghent University, Korte Meer 5, 9000 Gent, Belgium

## Abstract

**Background:**

Because numerous studies have revealed the negative consequences of stigmatisation, this study explores the determinants of stigma experiences. In particular, it examines whether or not part-time hospitalisation in contemporary psychiatric hospitals is associated with less stigma experiences than full-time hospitalisation.

**Methods:**

Survey data on 378 clients of 42 wards from 8 psychiatric hospitals are used to compare full-time clients, part-time clients and clients receiving part-time care as aftercare on three dimensions of stigma experiences, while controlling for symptoms, diagnosis and clients' background characteristics.

**Results:**

The results reveal that part-time clients without previous full-time hospitalisation report less social rejection than clients who receive full-time hospitalisation. In contrast, clients receiving part-time treatment as aftercare do not differ significantly from full-time clients concerning social rejection. No significant results for the other stigma dimensions were found.

**Conclusion:**

Concerning social rejection, immediate part-time hospitalisation could be recommended as a means of destigmatisation for clients of contemporary psychiatric hospitals.

## Background

Numerous public opinion studies have revealed the existence of negative stereotypes about persons with mental health problems [1-4]. They are typically considered to be dangerous, unpredictable or affected [5]. These negative stereotypes can have detrimental consequences for persons with mental health problems with regard to, for instance, employment and income [6], social integration [7] and adaptation [8], treatment continuation [9], medication adherence [10], life satisfaction [8,11,12], self-esteem [13] and symptoms [14]. Furthermore, stigma expectations can also lead persons to avoid or delay mental health treatment [15]. In addition to direct social rejection [16], more subtle mechanisms which produce the same outcomes have been studied. The modified labelling perspective, for example, states that negative societal attitudes become personally relevant and are translated into a concrete fear of devaluation and discrimination when persons receive psychiatric services [17,18]. This fear of devaluation and discrimination leads officially labelled persons to change their behaviour: they try to avoid actual negative reactions by means of secrecy or by avoidance of certain people. Fear of devaluation and discrimination and these adaptive strategies can have negative consequences for clients' self-concept and social relationships. Following the reasoning of the social stress perspective, which considers the self-concept and social support as important resources, both low self-esteem and self-efficacy, as well as poor social relationships, make (former) clients vulnerable for relapse. Thus, according to the modified labelling perspective, stigma expectations can produce negative consequences even if no direct social rejection or discrimination takes place.

The first studies about stigmatisation and its negative consequences appeared decades ago, when psychiatric treatment was predominantly long-term full-time hospitalisation in psychiatric hospitals [16]. Partially due to these kinds of studies, major changes in treatment have occurred that were assumed to be destigmatising. One of these changes is the rise of 'alternative' facilities. Another is the creation of more possibilities for short-term and part-time hospitalisation. Other studies have already compared stigma experiences between psychiatric hospitals and alternative settings such as psychiatric wards of general hospitals [19-21]. The empirical evidence of these studies is mixed: there is evidence both of more stigma related to alternative settings [19,20], and of more stigma related to psychiatric hospitals [19,21]. Two of these studies reveal that results can depend on the dimension of stigma under consideration [19,21].

In contrast with the empirical studies mentioned above, this study is not aimed at comparing stigma experiences between psychiatric hospitals and other types of organisations, but is interested in the role of part-time hospitalisation within psychiatric hospitals. Our main research question is whether differences in stigma could be found between part-time and full-time clients of contemporary psychiatric hospitals. This question is especially relevant in light of recent literature weighing the pros and cons of partial and full hospitalisation. A recent literature review revealed that outcomes of part-time clients are not different from those of full-time clients, but that the part-time clients and their families are more satisfied [22]. Therefore it is also interesting to explore the differences in stigma as an outcome as such, and because its relationship to clients' quality of life.

Three dimensions of stigma will be considered. *Stigma expectations *refer to clients' perceptions of the existence of negative attitudes in the general public, whereas *social rejection *and *self-rejection *refer to concrete experiences linked to clients' current treatment. Social rejection refers to negative reactions about the current treatment by people outside of the treatment setting. It should be conceptually distinguished from diminished social contacts, as it does not indicate the quantity but the quality of interaction, which is accentuated by pointing to negative reactions such as being treated with less respect. Self-rejection focuses on clients' negative reactions to themselves due to internalized stigma, such as feeling ashamed or inferior because of attendance of the current mental health service. It is important to mention that each of the three stigma concepts points out negative reactions to mental health service use and not negative reactions to the behaviour or symptoms of persons with mental health problems, as will be discussed in more detail when presenting the instruments. In this way the concepts explicitly refer to reactions to labels, in accordance with the conceptualisation of stigma by labelling theorists [e.g., [23]].

Which associations could be expected? For stigma expectations we expect no association, in accordance with the modified labelling perspective. Link argued that all persons in society get acquainted with the negative attitudes during their socialisation and that no systematic differences should be found between patients, former patients or the general public [17,18]. Following this reasoning, we have no reason to expect differences between patients who are hospitalised part-time or full-time.

Concerning social rejection, several hypotheses presenting the opposite perspective could be offered. On the one hand, we have reason to assume that partially hospitalised clients will be rejected less. They have a common structure to their daily lives, which offers more opportunity to hide the hospitalisation. This could make their hospitalisation less visible, when visibility is one of the key determinants of stigmatisation [24-26]. Furthermore, partial hospitalisation offers more opportunities to perform 'normal' roles outside the hospital, which are more highly valued than the 'psychiatric patient role'. Several authors have already explicitly mentioned alternative roles as a means to destigmatisation [27,28]. Finally, partially hospitalised patients could be considered as 'less sick' by their environment. In contrast with these three arguments, there are also reasons to expect that partially hospitalised clients will report more social rejection. As part-time clients spend more time in the community, they might be more exposed to negative reactions to their psychiatric hospitalisation, while full-time patients could be expected to be more protected from these reactions from outside the treatment setting. Therefore, our alternative hypothesis is that part-time psychiatric hospital patients report more stigma experiences.

Finally, we could follow a similar line of reasoning for self-rejection as was followed for social rejection. The larger opportunities to perform 'normal' roles outside the hospital and the clients' own perception of being 'less sick' could lead to less self-rejection for part-time hospitalised patients. However, the larger exposure to social rejection and to unintended stigmatising comments [29] could bring about more self-rejection.

One thing that should be taken into account is that part-time hospitalisation is often offered as aftercare to full-time hospitalisation, as a first step toward resuming the normal activities of daily life. As part-time clients in aftercare may receive certain reactions because of their former full-time hospitalisation, when studied they need to be distinguished from clients who are immediately placed in part-time hospitalisation.

Furthermore, it is likely that clients from the three groups differ from each other concerning for instance treatment history, diagnosis and symptom severity. Therefore, these characteristics are controlled for in this study. First, we account for symptoms, as clients with more symptoms may report more stigma experiences [30]. Second, we will include the psychiatric diagnosis, as stigma experiences can differ according to clients' diagnosis [31]. Furthermore, we consider the length of treatment as well as the total duration of treatment during clients' lives. Finally, we will add some socio-demographic variables as additional controls.

To summarize, this study intends to explore whether differences in three dimensions of stigma experiences could be found between full-time and part-time clients of contemporary psychiatric hospitals. Part-time treatment as aftercare will be distinguished from immediate part-time treatment.

## Methods

### Research design and sample

Our research question is investigated by comparing the self-reported stigma experiences of current clients of contemporary psychiatric hospitals. Survey data were used from a larger study on stigma experiences of clients from mental health services in Flanders, the northern part of Belgium, in 2005. In Belgium, psychiatric hospitals still play an important role in mental health care. Deinstitutionalisation started relatively late and Belgium still counts one of the largest numbers of psychiatric hospital beds per 100,000 people in Western Europe [32]. In Belgium, psychiatric treatment is mainly publicly funded but privately offered. Patients have a free choice of treatment and no obligatory catchment areas exist.

A clustered sample procedure was used to select the participants. From the total population of 41 psychiatric hospitals, 10 hospitals were randomly selected and invited to participate in the study. Due to two refusals because of reorganisations, the final sample consists of 8 hospitals. Not all wards of these hospitals were invited, however, because of the following criteria. First, wards exclusively for the young (< 18 years) or the old (> 60 or 65, according to the criteria of the hospital) were excluded. Second, wards which only had clients with cognitive disorders or mental retardation were excluded. Besides these two exclusion criteria concerning the wards, we also had exclusion criteria concerning the individual clients on the selected wards. Clients with mental retardation or cognitive disorders were excluded, as well as clients that were in an acute stage of illness (as determined by their nurse or physician in charge) and clients who did not have sufficient knowledge of Dutch to participate. The clients who fit the criteria and who were present on a date agreed upon beforehand were invited to participate. Anonymity in participation and confidentiality with regard to the use of the data collected were guaranteed. Clients were free to participate or not and could stop without giving any reason. The study was approved by the Ethical Committee of the Faculty of Political and Social Sciences of Ghent University. Informed consent was obtained after an introduction by the researcher. Of the 659 eligible clients, 445 (68%) agreed to participate. Our final sample thus consists of 445 clients from 44 wards from 8 psychiatric hospitals. As shown in the next section, the final working sample was slightly reduced further due to missing values.

### Measures

#### Dependent variables

*Stigma expectations *are measured by the Devaluation-Discrimination scale of Link [17,18]. It is composed of 12 items pointing to fear of devaluation and discrimination, and asks for clients' perceptions of how most people think of persons with mental health problems. The items are translated by the authors of the present study and slightly adapted by replacing the direct reference to psychiatric hospitalisation to 'persons who receive(d) psychological help', as suggested by Link et al. [33]. An example item is 'Most people would take them less seriously'. The scale has four answer categories from 'totally disagree' (1) to 'totally agree' (4). Although the original version of this scale is a 6-point scale, we decided to use the more recent 4-point version [e.g., [12,13]]. The scores are averaged to obtain a total score (M = 2.71; SD = 0.43; alpha = 0.84), with higher scores indicating more stigma expectations. *Social rejection *is measured by a Likert scale consisting of 5 items that is inspired by the social rejection subscale of Fife and Wright [34], and which indicates concrete negative reactions from the environment following mental health treatment. To explicitly refer responses to the immediate treatment context, the items are introduced by 'Since I come to this center' and by 'Some people react in a negative way toward themselves because they attend a center such as this one. Have you experienced the following reactions?' An example item is 'Since I come to this center, some people treat me with less respect.' The scale has five answer categories that are scored from 1 to 5 which are averaged to compute the total score (M = 3.21; SD = 1.23; alpha = 0.91), with higher scores indicating more social rejection. *Self-rejection *is analogously measured by a Likert scale consisting of 5 items that refer to negative self-evaluations that are linked to the attendance of the organisation. The items are inspired by the social isolation subscale of Fife and Wright [34] and are introduced by a sentence that makes explicit that the current treatment is stressed. An example item is 'Since I come to this center, I have come to feel inferior.' The items are coded from 'totally disagree' to 'totally agree', with scores from 1 to 5, with higher scores indicating more self-rejection. The total self-rejection score is computed as the mean score on the 5 items (mean = 2.86; SD = 1.28; alpha = 0.91).

#### Independent variables

Three types of hospitalisation are considered: full-time hospitalisation, immediate part-time hospitalisation and part-time hospitalisation as aftercare. We constructed two dummy variables with full-time hospitalisation as a reference category. *Part-time hospitalisation *refers to partial hospitalisation that is not preceded by full-time hospitalisation, whereas *part-time hospitalisation as aftercare *was preceded by full-time hospitalisation. *Full-time hospitalisation *means that clients stay day and night, whereas *part-time *refers to a part of the day. In most cases, it means that clients live at home and attend the hospital by day for therapy sessions. There are also some patients living in the hospital and working part-time, but this form of part-time hospitalisation is rare. As the number of clients in our sample hospitalised part-time is not large, we could not differentiate between these two forms. *Symptoms *are measured by the Brief Symptom Inventory-18 (BSI-18) [35], using the Dutch translation of the items of the SCL-90-R [36]. It is a Likert scale consisting of 18 items with scores from 0 to 4. These scores are averaged to obtain a total symptoms score, with higher scores indicating more symptoms (mean = 1.36; SD = 0.97; alpha = 0.95). *Length of stay *of the current treatment is measured in months. The *length of treatment history *is computed as the difference between current age and the age at which one first received professional mental health care. Diagnostic information was obtained anonymously from the nurse or physician in charge of the client. Three main diagnostic categories were used as dichotomous variables in the analysis (1 = present, 0 = absent) to measure *diagnosis*: mood disorders, psychotic disorders and substance-related disorders. Finally, the following socio-demographic characteristics are also used as controls: *gender *(men = 2, women = 1), *age *(in years) and *marital status *(married or cohabiting = 1, single, divorced or widowed = 0). *Income *is measured by a proxy indicating how easily one gets by on a monthly income (from 1 = very difficultly to 6 = very easily). *Education *is measured by four categories (from 1 = primary degree to 4 = university).

## Results

### Description of the sample

The final working sample (Table 1) consists of 378 clients because of missing values for 67 clients (15%) on some key variables. For instance, information concerning self-rejection and social rejection was missing for 22 clients, concerning stigma expectations for 16 clients. In the final working sample, age varies from 16 to 73, with an average age of 38, 48.1% are men, and 23% of the respondents are married or cohabiting. Concerning their education, 3.4% have finished primary education (up to 12 years of age), 20.6% the first three years of secondary education (up to 15 years), 53.4% the last three years of secondary education (up to 18 years), and 22.5% have finished college (up to 21 or 22 years). Regarding the main diagnostic categories, 27% of the clients have a mood-related disorder, 24.1% a psychotic disorder and 33.3% a substance-related disorder. The other clients have a large diversity of other diagnoses, which are not analysed separately in this study, as mentioned above in the description of the variables. The mean level of symptoms as measured on the BSI-18 with a range from 0 to 4 is 1.36, with a standard deviation of 0.97. The mean length of current treatment is 16 months, the average number of years since first treatment is 10 years. The majority of the clients were hospitalised full-time: 226 out of 378 (59.8%). Of the clients, 72 were immediately hospitalised part-time (19%) and 80 were hospitalised part-time as aftercare (21.2%).

**Table 1:** Description of the sample

	Total sample (N = 378)	Immediate part-time hospitalisation (N = 72)	Part-time hospitalisation aftercare (N = 80)	Full-time hospitalisation (N = 226)	P difference between groups^1^
Gender (% men)	48.10	40.30	52.50	49.10	0.290
Age (mean, SD)	38.28 (12.02)	36.60 (10.57)	44.66 (11.55)	36.56 (11.89)	0.000
Education (mean, SD)	2.95 (0.75)	3.06 (0.84)	2.91 (0.68)	2.93 (0.75)	0.411
Marital status (% married or cohabiting)	22.75	23.60	30.00	19.90	0.177
Income (mean, SD)	3.34 (1.43)	3.40 (1.49)	3.60 (1.37)	3.23 (1.42)	0.128
Symptoms (mean, SD)	1.36 (0.97)	1.38 (1.05)	1.30 (0.99)	1.38 (0.94)	0.804
Length of stay, in months (mean, SD)	16.08 (32.55)	11.70 (21.82)	33.69 (43.50)	11.25 (28.65)	0.000
Length treatment history, in years (mean, SD)	10.18 (8.75)	9.49 (8.38)	13.93 (9.47)	9.07 (8.26)	0.000
% Mood-related disorder	26.98	33.30	26.30	25.20	0.396
% Psychotic-related disorder	24.07	27.80	25.00	22.60	0.651
% Substance-related disorder	33.33	16.70	31.30	39.40	0.002

^1 ^Based on chi-square test for comparison of percentages; ANOVA for comparison of means.

Table 1 also provides more information concerning the similarities and differences between the three groups. These results reveal no differences concerning gender, education, marital status, income and level of symptoms. Concerning the diagnosis, the results reveal that clients that receive immediate part-time hospitalisation have a substance-related disorder less often. Furthermore, the results reveal several distinctions between clients that are hospitalised part-time as aftercare and the other two groups: they are older, they have a longer previous treatment history and they have a longer current treatment duration. It is important to take these significant differences into account when comparing stigma experiences between these three groups.

### Results of the regression analysis

The data are analysed by means of ordinary least squares regression analysis for each stigma variable separately, using SPSS 12.0. Before answering our research questions by means of these regression analyses, however, the relationships between the independent variables are explored (Table 2). As the highest intercorrelation between two variables that appear together as predictor variables in the analysis is 0.460 (the correlation between age and length of treatment history), we do not expect problems with multicollinearity.

**Table 2:** Bivariate correlations between independent variables: Pearson's correlation coefficients and *p *values; N = 378

	1.	2.	3.	4.	5.	6.	7.	8.	9.	10.	11.	12.	13.
1. Immediate part-time hospitalisation	1.000												
2. Part-time hospitalisation aftercare	-0.251												
	*0.000*												
3. Full-time hospitalisation	-0.588	-0.615											
	*0.000*	*0.000*											
4. Gender	-0.076	0.045	0.029										
	*0.138*	*0.382*	*0.577*										
5. Age	-0.068	0.276	-0.184	-0.103									
	*0.187*	*0.000*	*0.000*	*0.046*									
6. Education	0.068	-0.026	-0.041	-0.090	0.012								
	*0.186*	*0.619*	*0.429*	*0.079*	*0.810*								
7. Marital status	0.010	0.090	-0.080	-0.245	0.276	-0.014							
	*0.847*	*0.082*	*0.122*	*0.000*	*0.000*	*0.785*							
8. Income	0.021	0.094	-0.090	0.105	0.131	-0.004	0.069						
	*0.686*	*0.068*	*0.081*	*0.041*	*0.011*	*0.942*	*0.180*						
9. Symptoms	0.008	-0.034	0.017	-0.319	-0.097	-0.006	0.072	-0.284					
	*0.870*	*0.508*	*0.748*	*0.000*	*0.061*	*0.905*	*0.160*	*0.000*					
10. Length treatment history	-0.039	0.223	-0.154	-0.154	0.460	-0.038	0.100	0.036	0.008				
	*0.454*	*0.000*	*0.003*	*0.003*	*0.000*	*0.458*	*0.052*	*0.480*	*0.869*				
11. Mood-related disorder	0.069	-0.009	-0.045	-0.252	0.107	0.064	0.182	-0.026	0.183	0.071			
	*0.178*	*0.868*	*0.382*	*0.000*	*0.038*	*0.212*	*0.000*	*0.610*	*0.000*	*0.170*			
12. Psychotic-related disorder	0.042	0.011	-0.040	0.238	-0.089	-0.012	-0.128	0.084	-0.218	0.030	-0.287		
	*0.415*	*0.828*	*0.439*	*0.000*	*0.084*	*0.820*	*0.012*	*0.102*	*0.000*	*0.559*	*0.000*		
13. Substance-related disorder	-0.171	-0.023	0.160	0.228	0.138	0.017	-0.076	-0.012	-0.220	-0.070	-0.303	-0.359	
	*0.001*	*0.657*	*0.002*	*0.000*	*0.007*	*0.736*	*0.141*	*0.819*	*0.000*	*0.175*	*0.000*	*0.000*	
14. Length of stay	-0.065	0.281	-0.181	0.004	0.210	-0.089	0.014	0.137	-0.016	0.286	-0.027	0.186	-0.159
	*0.204*	*0.000*	*0.000*	*0.940*	*0.000*	*0.086*	*0.783*	*0.007*	*0.751*	*0.000*	*0.604*	*.000*	*0.002*

What did the results reveal (Table 3)? As expected, no significant association with part-time hospitalisation was found for stigma expectations. As predicted by the principles of the modified labelling perspective, clients in immediate part-time treatment or as aftercare do not have less stigma expectations (B = 0.059; SE = 0.054; p = 0.281 and B = 0.028; SE = 0.055; p = 0.609, respectively). In contrast, one significant negative association was found for social rejection. Clients who are immediately hospitalised part-time report less social rejection in comparison with full-time clients (B = 0.304; SE = 0.154; p = 0.049). Clients receiving part-time hospitalisation as aftercare do not differ significantly from full-time clients however (B = 0.213; SE = 0.156; p = 0.174). Concerning self-rejection, no significant results were found. There was no association with part-time hospitalisation as aftercare (B = 0.031; SE = 0.156; p = 0.842). The link with immediate part-time treatment was negative and nearly significant however (B = -0.288; SE = 0.153; p = 0.061) and is in the same direction as for social rejection.

**Table 3:** The association between treatment and client characteristics and three dimensions of stigmatisation.

	Stigma expectations				Social rejection				Self-rejection			
	B	SE	Beta	sig	B	SE	Beta	sig	B	SE	Beta	sig
Constant	2.650	0.153		0.000	2.955	0.433		0.000	2.169	0.432		0.000
Immediate part-time hospitalisation	0.059	0.054	0.053	0.281	-0.304	0.154	-0.097	0.049	-0.288	0.153	-0.089	0.061
Part-time hospitalisation aftercare	0.028	0.055	0.027	0.609	0.213	0.156	0.071	0.174	0.031	0.156	0.010	0.842
Full-time hospitalisation (ref)												
Gender	-0.034	0.047	-0.039	0.470	-0.077	0.134	-0.032	0.563	-0.126	0.133	-0.049	0.345
Age	-0.002	0.002	-0.063	0.279	0.003	0.006	0.026	0.658	0.013	0.006	0.127	0.024
Education	0.017	0.027	0.029	0.535	-0.089	0.077	-0.055	0.248	0.032	0.077	0.019	0.674
Marital status	0.033	0.052	0.032	0.533	0.100	0.148	0.034	0.502	0.159	0.148	0.052	0.281
Income	-0.042	0.015	-0.140	0.005	-0.126	0.043	-0.147	0.003	-0.069	0.043	-0.077	0.108
Symptoms	0.158	0.024	0.355	0.000	0.462	0.068	0.366	0.000	0.598	0.068	0.456	0.000
Length treatment history	0.004	0.003	0.081	0.143	0.008	0.008	0.056	0.305	-0.024	0.008	-0.162	0.002
Mood-related disorder	0.000	0.054	0.000	1.000	0.105	0.154	0.038	0.497	-0.371	0.154	-0.129	0.016
Psychotic-related disorder	-0.023	0.062	-0.023	0.706	0.573	0.175	0.200	0.001	0.189	0.175	0.063	0.280
Substance-related disorder	0.018	0.058	0.019	0.763	0.187	0.165	0.072	0.259	-0.181	0.165	-0.067	0.273
Length of stay	0.000	0.001	0.031	0.550	0.000	0.002	0.002	0.973	0.003	0.002	0.076	0.128

R^2^	0.204				0.207				0.273			

Results of an ordinary least squares regression analysis (N = 378)

It is important to mention that these results are found controlling for symptoms, treatment history, diagnosis and other background characteristics, as the comparison in Table 1 reveals differences between the three groups concerning age, previous treatment history, current length of stay and the percentage of clients with a substance-related disorder. Concerning these differentiating variables, the regression analysis reveals that both age and previous treatment history are linked with self-rejection: older persons report more self-rejection, while persons with a longer treatment history report less.

## Discussion

The evidence about stigmatisation of persons with mental health problems and its negative consequences constituted one of the reasons major reforms in mental health care during the last several decades occurred. In contrast with the prevalence of long-term full-time hospitalisation in psychiatric hospitals decades ago, emphasis is now placed on providing more alternative facilities on the one hand and on offering more short-term and part-time hospitalisation on the other. As several studies have already focused on the comparison of alternative facilities with the traditional psychiatric hospitals, this article aimed at comparing full-time with part-time hospitalisation in contemporary psychiatric hospitals.

Before discussing the results, some shortcomings of the study should be mentioned. A first limitation concerns the participation in the study. Besides a general non-response rate of 32%, some additional cases were dropped due to incomplete administering of the survey. Concerning both the general non-response and a large part of the additional dropouts, because we have per definition no information on these clients' stigma experiences it is not clear in what way this may have biased our findings. It is for instance possible that clients with more stigma expectations or concrete stigma experiences were more inclined to refuse to participate or to drop out during their participation. This would imply that the mean level of stigma in our study is underestimated. Unfortunately, data about the reasons for refusal or dropout were not available, as the ethical considerations of this study allowed clients to refuse or to withdraw from participation without giving any reason. As official statistics of the intended target group were not available, we did not have the option of comparing the characteristics of the participating clients with the total group. For the same reason we also do not have information about the kind of hospitalisation (full-time or part-time) of the clients that did not participate, which also limits our ability to estimate the representativeness of the three groups in our study.

Furthermore, the exclusion of patients in a too acute stage of illness also limits the generalisability of this study. It could be expected that the symptom levels of the participants are rather moderate in comparison with the clients who were excluded because of their too acute stage of illness. This would mean that the mean level of symptoms is underestimated. Concerning the self-reports about stigma, one could assume that the exclusion of the most acute clients adds to the internal validity of measuring stigma, as clients in an acute phase could be expected to give less valid accounts of their stigma experiences as their experiences might be symptom related. Furthermore, the concept of stigma might be experienced as being too abstract or as being too painful for clients in such an acute phase. However, the exclusion of these clients limits the external validity by reducing the representativeness of the study to all clients of psychiatric hospitals.

A second shortcoming concerns the research design: there is no controlled allocation of clients to the three treatment conditions and no longitudinal data are available. This shortcoming reduces our ability to rule out alternative causal paths. Clients could for instance choose a certain treatment type for fear of stigma. However, a significant relationship between treatment type and stigma expectations would then be expected, which was not the case. Furthermore, social rejection and self-rejection refer to stigma experiences which occur after the clients have entered the current treatment setting. For these reasons, we do not assume that this alternative causal path is very plausible. However, other (self-)selection mechanisms could have been at work. It is for instance possible that clients with more severe symptoms are systematically referred for full-time hospitalisation. Several client background variables were included in the analyses to account for this possibility, including a measure of symptoms and diagnosis. Whereas several potentially relevant differences between the clients are already taken into account, it must be admitted that not all potential confounders could be controlled for, however.

A third limitation concerns the conceptualisation of stigmatisation. Two dimensions of it – social rejection and self-rejection – point to the current treatment period. This is an important quality for estimating the link with characteristics of clients' current treatment. However it is possible that stigma experiences due to current treatment are affected by clients' previous treatment history. As the results of the multilevel analyses reveal, clients with a longer treatment history do report less self-rejection. As clients from the three treatment conditions differ concerning length of treatment history, it was important to account for this in the analysis. However, our measure of the treatment history captures only its length. It is likely that stigma experiences which occur because of current treatment are also determined by the extensiveness of the previous treatment. Unfortunately, no such measure was available. Another limitation applies to the specific measure of social rejection. In this study social rejection is conceptualised as negative reactions to current mental health treatment from people outside of the treatment setting. However, both classic and recent studies have mentioned that clients also experience negative reactions from people within the setting, such as staff members and other clients [e.g., [16,37]]. Concerning the types of treatment under study, it is possible that full-time clients also experience more social rejection from the other actors within the treatment context as they spend more time with them. Therefore, an interesting elaboration of this study would be to extend the study to include the other actors within the treatment context. Furthermore, although the measures explicitly refer to stigma associated with the treatment context and although symptoms are controlled for in the analyses, the possibility that clients consider negative reactions to their symptoms rather than to their psychiatric hospitalisation as stigma cannot be completely ruled out.

Fourth, the extent of the association between part-time hospitalisation and social rejection was rather small. The relationship was significant however, despite the small sample size, especially concerning the two part-time hospitalisation groups. The association with self-rejection was nearly significant but in the same direction. As this lack of strong results could be partially due to the small number of part-time hospitalisations in our sample, it would be interesting to replicate this kind of analysis with larger samples. Larger samples would also allow researchers to differentiate between so-called 'day patients' and 'night patients'. This would be especially interesting as our study only differentiated between two types of part-time hospitalisation. There are additional reasons to further refine this kind of analysis in future research. For instance, as Kallert et al. reveal [38], a large diversity of types of day hospitalisation exists between and within countries in Europe. Furthermore, a recent literature review about the pros and cons of partial hospitalisation suggests that more attention be paid to its different types [22]. Another related suggestion is that further research be done to find out whether our results could be replicated in other countries. This is important not only in light of the large diversity of day hospitalisation types in Europe, it is also important because this study is limited to a country where psychiatric hospitals are still playing an important role. Therefore, we suggest replicating this kind of study in countries with a more community-based mental health system. It might be particularly interesting to compare stigma experiences of former full-time and part-time hospital patients once they have recovered.

Despite these shortcomings, we believe that this study has yielded some interesting findings. The analysis showed that part-time clients report less social rejection in comparison with clients who are hospitalised full-time. Possible explanations are that they have more possibilities to hide their hospitalisation, more opportunities to perform 'normal', highly valued roles and are considered as being less 'sick'. Our alternative hypothesis, which predicted that clients with more exposure to the community will be rejected more, is not supported by our data.

An important finding is that the association is only negative for clients who were not formerly hospitalised full-time. Clients in part-time treatment as a transition to discharge are *not *rejected less by their environment. This result implies that they may be judged more in accordance with their former situation as a full-time client than with their present status as a part-time client. This means that this key idea about stigmatisation, that persons are judged more on their past rather than their present situation, seems to apply even for clients who are still hospitalised. Similarly to former clients who are evaluated on their previous hospitalisation, current part-time clients seem to be evaluated in accordance with their previous full-time hospitalisation. Therefore, these results seem to suggest that part-time hospitalisation can only contribute to destigmatisation if it is not preceded by full-time hospitalisation.

## Conclusion

To conclude, our results concerning social rejection suggest that immediate part-time hospitalisation could be recommended as a means to destigmatisation for current clients of psychiatric hospitals, as far as is possible in the light of patients' conditions. Although the long-term solution for the stigma problem is a change in the negative attitudes of the general public, other studies have already revealed that these attitudes are not easily changed [e.g., [3]]. Therefore, in anticipation of large-scale and long-term changes at the level of the general public, actions at the level of the organisation of mental health care should be paid more attention to. For those clients currently receiving mental health treatment, this kind of effort could already make a difference.

## Competing interests

The authors declare that they have no competing interests.

## Authors' contributions

MV was responsible for the general research design, data collection and analysis. She prepared the first draft of the article. PB contributed to the general research design and critically revised the first draft. WC contributed to the first draft and the critical revision of the manuscript. All authors read and approved the final manuscript.

## Pre-publication history

The pre-publication history for this paper can be accessed here:


